# The relationship between different types of caries and periodontal disease severity in middle-aged and elderly people: findings from the 4th National Oral Health Survey of China

**DOI:** 10.1186/s12903-021-01585-1

**Published:** 2021-05-03

**Authors:** Li Xia Yu, Xing Wang, Xi Ping Feng, Bao Jun Tai, De Yu Hu, Bo Wang, Chun Xiao Wang, Shu Guo Zheng, Xue Nan Liu, Wen Sheng Rong, Wei Jian Wang, Yan Si, Huan Cai Lin

**Affiliations:** 1 Hospital of Stomatology, Guanghua School of Stomatology, Sun Yat-Sen University, 56 West Lingyuan Road, Guangzhou, 510000 People’s Republic of China; 2 Guangdong Provincial Key Laboratory of Stomatology, Guangdong Key Laboratory for Dental Disease Prevention and Control, Institute of Stomatology, Sun Yat-Sen University, Guangzhou, People’s Republic of China; 3 Chinese Stomatological Association, Beijing, People’s Republic of China; 4 Shanghai Ninth People’s Hospital, Shanghai JiaoTong University School of Medicine, Shanghai, People’s Republic of China; 5 School and Hospital of Stomatology, Wuhan University, Wuhan, People’s Republic of China; 6 West China Hospital of Stomatology Sichuan University, Chengdu, People’s Republic of China; 7 Chinese Center for Disease Control and Prevention, Beijing, People’s Republic of China; 8 Peking University School and Hospital of Stomatology, 22# Zhongguancun South Avenue, HaiDian District, Beijing, 100081 People’s Republic of China

**Keywords:** Dental caries, Periodontal disease, Epidemiology

## Abstract

**Background:**

The relationship between dental caries and periodontal disease is still controversial. The objective of this study was to explore the relationship between different types of caries and periodontal disease severity in middle-aged and elderly people in China.

**Methods:**

The study population consisted of 4407 middle-aged and 4117 elderly subjects. Caries were divided into the following three types: type A, crown caries; type B, lesions involving both the crown and root, representing mixed-type caries; and type C, root caries. These three types together represent the overall caries situation, which we call type ABC. Caries were quantitated by decayed and filled teeth (DFT index). Periodontitis was evaluated by clinical attachment loss.

**Results:**

Middle-aged people with periodontitis had a significant association with DFT type B (OR: 1.21, 95% CI 1.17–1.25) and type C (OR: 1.40, 95% CI 1.24–1.56). Elderly people with periodontitis had a significant association with DFT type C (OR: 1.28, 95% CI 1.21–1.35).

**Conclusions:**

In China, caries types B/C were positively correlated with periodontitis in the middle-aged group, and only caries type C was positively correlated with periodontitis in the elderly group.

## Background

Dental caries and periodontitis are the most common diseases in the oral cavity among adults. Both diseases are the primary cause of tooth loss and can lead to negative impacts on quality of life. To date, the relationship between the two diseases is still controversial. Although several studies have shown a positive correlation between the two diseases [1–10], some studies have shown a negative correlation [11–13] or no association [14].

The reasons for the positive correlation include common social-behavioural factors, such as age, sex, poor oral hygiene, and smoking status [15]. The main reason for the inverse correlation is different bacteriological spectra. Accumulating evidence suggests that *Streptococcus mutans* is the primary cariogenic bacterium, while periodontitis is associated with specific gram-negative anaerobic bacteria, such as *Porphyromonas gingivalis*. A negative association has been reported between salivary levels of *S. mutans* and *P. gingivalis* in subjects with varying severities of caries and periodontitis, indicating that an inverse correlation exists between the two diseases [13].

Caries can be divided into the following three types according to the location of occurrence: crown caries, root caries and mixed-type caries affecting both the crown and root. According to WHO guidelines, if a carious lesion involves both the crown and the root, the possible site of origin of the carious lesion should be recorded as the site of decay. When it is impossible to determine the origin of the lesion, both the crown and root should be recorded as decayed [16]. In fact, in a large-scale oral epidemiological examination, confirming the origin of caries was difficult in most cases. Therefore, classifying caries involving both the crown and root as coronal caries or root caries is challenging. To the best of our knowledge, regarding the relationship between the two diseases, few studies have been able to clearly identify the specific types of caries studied [1, 7]. Different caries detection methods could be a plausible explanation for the discrepancies found in the relationship between caries and periodontitis [12]. Due to the controversial relationship between the two diseases, it is necessary to classify caries.

Inconsistencies in subject selection could be another plausible explanation for the discrepancies found between the two diseases [12]. Studies reporting an inverse association between the two diseases are based mostly on selected patients who constitute a young population no older than 20 years with juvenile periodontitis, who have been recognized as having increased susceptibility to periodontitis [14]. Similar to many diseases, age is an important factor that has a greater impact than other known risk factors on caries and periodontitis and can explain the variation in occurrence. The role of age in both diseases has been attributed to accumulated exposure. In addition, a recent study showed that susceptibility to the two diseases could change with ageing [17].

Taken together, these findings show that the relationship between dental caries and periodontal disease remains controversial. No large sample studies have investigated the relationship between these two diseases in the Chinese population. The objective of this study was to explore the relationship between different types of caries and periodontal disease severity while considering different types of caries in middle-aged and elderly people from the 4th National Oral Health Survey in China. As caries and periodontitis are related to many social and behavioural background factors, ordered logistic regression was used to control for relevant confounders.

## Methods

### Sampling and clinical examination method

The present study was a part of the 4th National Oral Health Survey, which is the most comprehensive oral health survey to date in China, and was carried out during 2015–2016. A multistage stratified sampling method was used to recruit adults from all 31 provinces, municipalities and autonomous regions in mainland China. The probability proportional to size (PPS) method was used to randomly select subjects [18]. In total, 4410 35- to 44-year-olds and 4431 65- to 74-year-olds completed the survey. The exclusion criteria for this analysis were participants who were edentulous and had a periodontal status that could not be examined for any reason, such as a nonstandard fixed prosthesis that covered the gingiva or the presence of calculus to such an extent that a periodontal examination was impossible.

The latest WHO recommendations (2013) with appropriate adjustments according to the actual situation were used for the clinical oral examinations. The examinations were conducted by three trained licenced dentists, while three other trained individuals with clinical experience acted as recorders in each province. The examinations were conducted with a mobile dental chair using artificial light, a disposable dental mirror, and a standard WHO Community Periodontal Index (CPI) probe [18, 19].

### Variables

#### Independent variables

**Fig. 1:**
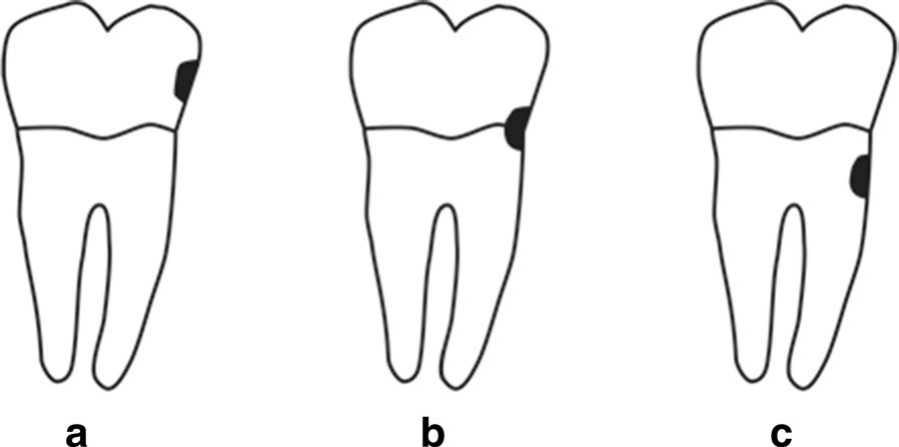
Types of dental caries according to location Type A, caries or a filling on the crown, representing crown caries. Type B, caries or a filling involving both the crown and root, representing mixed-type caries. Type C, caries or a filling on the root, representing root caries. These three types together represent the overall caries situation, which we call type ABC

Caries were recorded for all tooth surfaces, but the observations were recorded for each tooth. We divided the caries into the following three types: type A, caries or a filling on the crown, representing crown caries; type B, caries or a filling involving both the crown and root, representing mixed caries; and type C, caries or a filling on the root, representing root caries. These three types together represent the overall caries situation, which we call type ABC (Fig. 1). Because residual roots involve both the crown and root, we classified residual roots as type B. Missing teeth of any cause in adults were recorded as code 5 in our national survey, which differs from the WHO recommendations. The number of decayed and filled teeth (DFT) was calculated to analyse its relationship with periodontitis. The kappa value of the inter-examiners’ reliabilities in the examination of dental caries was 0.97 in both the middle-aged and elderly groups [18, 20].

#### Dependent variables

Periodontal disease severity was evaluated by clinical attachment loss (CAL) in each tooth. A full mouth examination including the third molars was performed. The tooth with the worst CAL score was recorded for the person-level score. Probing was performed by “walking around” along gingival crevices with a standardized force of no more than 20 g. Each tooth was recorded according to its severity. CAL was scored as 0 (0–3 mm), 1 (4–5 mm), 2 (6–8 mm), 3 (9–11 mm), 4 (12 mm or more), 9 (tooth excluded) or X (tooth not present). For the analysis, periodontitis was divided into the following three groups according to CAL: ≤ 3 mm, 4–5 mm, and ≥ 6 mm. Before the field investigation, the examiners underwent training, and each examiner and reference examiner performed an examination of 10 to 15 subjects per group to assess their consistency. The kappa value was calculated to be greater than 0.6, suggesting good reliability [18].

#### Covariates

The covariates included in the statistical analysis were social economic status (SES), such as sex (female or male), area (urban or rural), education level (subjects were classified according to whether they had received nine-year compulsory education and were divided into groups with ≤ 9 years or > 9 years), and household income per capita (less than RMB 5000/person, RMB 5000 to RMB 15,000/person, more than RMB 15,000/person, or prefer not to answer); oral health-related behaviours, such as the frequency of dessert consumption (< twice a day or ≥ twice a day), frequency of tooth brushing (< once per day or ≥ once per day), use of dental floss (no or yes), use of a toothpick (no or yes), smoking status (never or current), and alcohol consumption (ceased, rarely/never, daily, or weekly); and diabetes history (no or yes) [21, 22].

### Statistical analysis

The data analysis was carried out using SPSS 20.0. Chi-square tests were performed to compare periodontitis according to the participant characteristics. To determine the association between periodontitis and dental caries, ordinal logistic regression models were used. First, a bivariate analysis was performed. Then, the independent variables with *P* ≤ 0.25 based on the bivariate logistic analysis were further tested in the multivariate analysis. Three ordinal logistic regression models were constructed to measure the crude and adjusted effects of the DFT scores on periodontitis. In Model 1, the DFT score was introduced as the only independent variable. Then, in Model 2, the DFT score and SES were included. Finally, the DFT score, SES, oral health-related behaviours and diabetes were included in Model 3. A *P*-value < 0.05 in all two-sided statistical tests was considered significant. The statistical analyses did not include missing values or individuals who preferred not to answer.

## Results

**Fig. 2:**
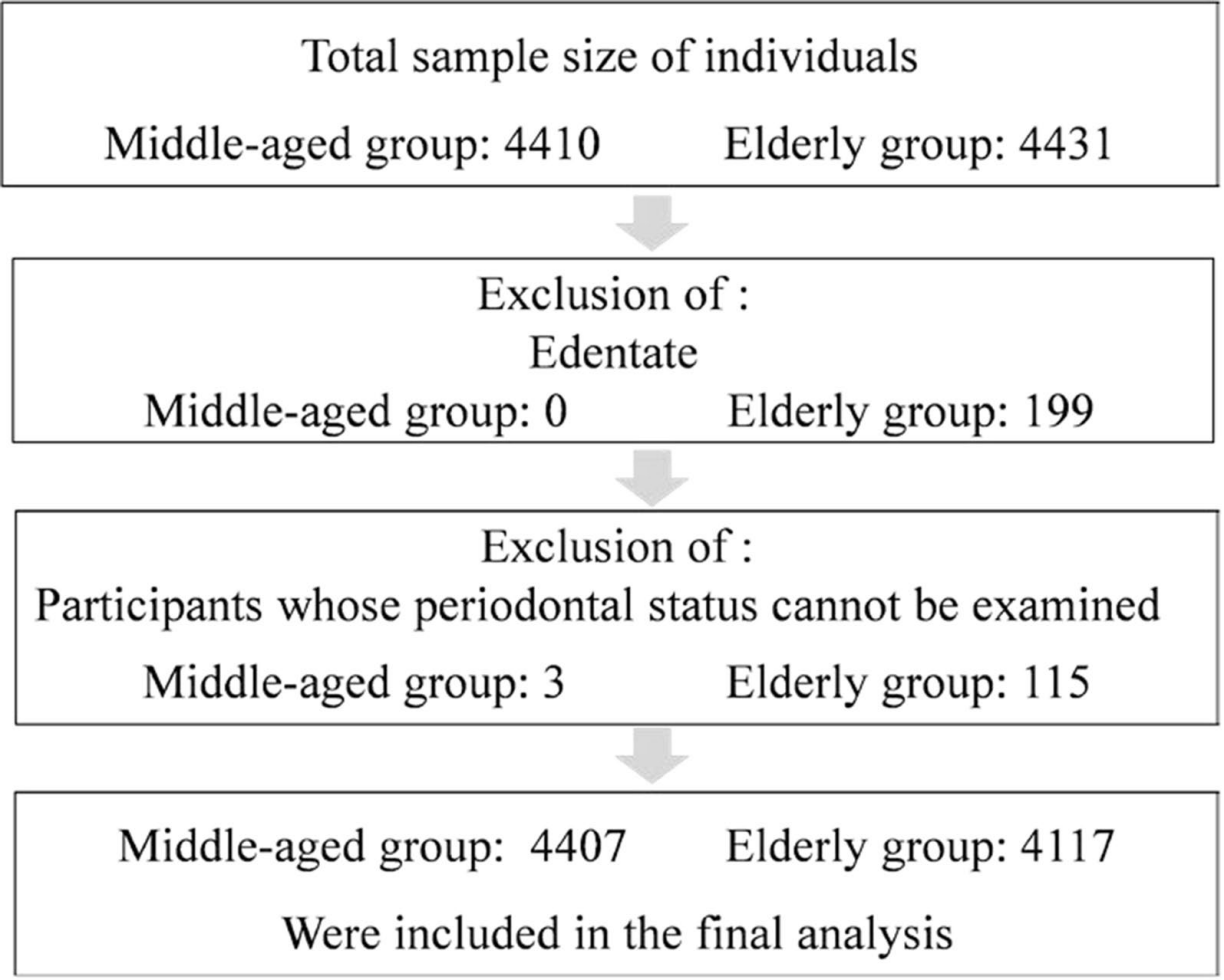
Flow chart of data collection

In total, 4410 subjects aged 35–44 years and 4431 subjects aged 65–74 years completed the national survey. Participants who were edentulous were excluded as follows: subjects aged 35–44 years (n = 0) and subjects aged 65–74 years (n = 199, 4.49%). The participants were excluded if their periodontal status could not be examined for any reason as follows: subjects aged 35–44 years (n = 3, 0.07%) and subjects aged 65–74 years (n = 115, 3.66%). Finally, a sample of 4407 subjects aged 35–44 years and 4117 subjects aged 65–74 years were included in the analysis (Fig. 2).

Table 1 shows the results of the bivariate analysis of characteristics of participants in the 35- to 44-year-old group in relation to their periodontal status. We found that sex, area, education level, household income per capita, toothbrushing frequency, use of dental floss, use of a toothpick, smoking status, alcohol consumption, diabetes and caries types (A/B/C/ABC) were associated with periodontal disease severity (*P* ≤ 0.25). Table 2 shows the results of the bivariate analysis of characteristics of participants in the 65- to 74-year-old group in relation to their periodontal status. We found that sex, area, education level, household income per capita, toothbrushing frequency, use of dental floss, use of a toothpick, smoking status, alcohol consumption and caries type (A/B/C/ABC) were associated with periodontal disease severity (*P* ≤ 0.25).

**Table 1:** Bivariate analysis of characteristics of participants in the 35- to 44-year-old group in relation to their periodontal status

Variable	Overall	Degree of periodontitis			*P *value^a^
		CAL ≤ 3 mm	CAL = 4–5 mm	CAL ≥ 6 mm	
Total, n (%)	4407 (100.0)	2944 (66.8)	1124 (25.5)	339 (7.7)	
Socioeconomic characteristics					
Sex, n (%)					< 0.001
Female^†^	2212 (50.2)	1602 (54.4)	505 (44.9)	105 (31.0)	
Male	2195 (49.8)	1342 (45.6)	619 (55.1)	234 (69.0)	
Missing values					
Area, n (%)					0.001
Urban^†^	2089 (47.4)	1454 (49.4)	492 (43.8)	143 (42.2)	
Rural	2318 (52.6)	1490 (50.6)	632 (56.2)	196 (57.8)	
Missing values					
Educational level, n (%)					< 0.001
≤ 9 years^†^	3131 (71.0)	1983 (67.4)	878 (78.1)	270 (79.6)	
> 9 years	1276 (29.0)	961 (32.6)	246 (21.9)	69 (20.4)	
Missing values					
Household income per capita, n (%)					< 0.001
.Less than RMB 5000/person^†^	924 (21.0)	551 (18.7)	276 (24.6)	97 (28.6)	
RMB 5000 to RMB 15,000/person	1357 (30.8)	912 (31.0)	336 (29.9)	109 (32.2)	
More than RMB 15,000/person	1391 (31.6)	982 (33.4)	319 (28.4)	90 (26.5)	
Prefer not to answer ^c^	735 (16.7)	499 (16.9)	193 (17.2)	43 (12.7)	
Missing values					
Oral health-related behaviours					
Toothbrushing frequency, n (%)					< 0.001
< Once per day^†^	4110 (93.3)	2808 (95.4)	1009 (89.8)	293 (86.4)	
≥ Once per day	297 (6.7)	136 (4.6)	115 (10.2)	46 (13.6)	
Missing values					
Use of dental floss, n (%)					< 0.001
No^†^	4176 (94.8)	2761 (93.8)	1087 (96.7)	328 (96.8)	
Yes	230 (5.2)	183 (6.2)	36 (3.2)	11 (3.2)	
Missing values	1 (0)	0 (0)	1 (0.1)	0 (0)	
Use of a toothpick, n (%)					< 0.001
No^†^	2395 (54.3)	1679 (57.0)	567 (50.4)	149 (44.0)	
Yes	2012 (45.7)	1265 (43.0)	557 (49.6)	190 (56.0)	
Missing values					
Frequency of dessert consumption, n (%)					0.733
< Twice a day^†^	1005 (22.8)	681 (23.1)	247 (22.0)	77 (22.7)	
≥ Twice a day	3402 (77.2)	2263 (76.9)	877 (78.0)	262 (77.3)	
Missing values					
Smoking status, n (%)					< 0.001
Never^†^	1456 (33.0)	862 (29.3)	435 (38.7)	159 (46.9)	
Current (former)	2951 (67.0)	2082 (70.7)	689 (61.3)	180 (53.1)	
Missing values					
Alcohol consumption, n (%)					0.039
Ceased^†^	206 (4.7)	128 (4.3)	59 (5.2)	19 (5.6)	
Rarely/never	373 (8.5)	230 (7.8)	106 (9.4)	37 (10.9)	
Daily	3710 (84.2)	2515 (85.4)	927 (82.5)	268 (79.1)	
Weekly	115 (2.6)	68 (2.3)	32 (2.8)	15 (4.4)	
Missing values ^b^	3 (0.1)	3 (0.1)	0 (0)	0 (0)	
Systemic diseases associated with periodontitis					
Diabetes, n (%)					< 0.001
No^†^	4319 (98.0)	2900 (98.5)	1099 (97.8)	320 (94.4)	
Yes	88 (2.0)	44 (1.5)	25 (2.2)	19 (5.6)	
Missing values					
Caries type, n (%; 95% CI)					
Type A	2442 (55.4; 53.9–56.9)	1590 (54.0; 52.2–55.8)	652 (58.0; 55.1–60.9)	200 (59.0; 53.6–64.3)	0.028
Type B	1043 (23.7; 22.4–25.0)	560 (19.0; 17.6–20.5)	331 (29.4; 27.8–32.2)	152 (44.8; 39.5–50.3)	< 0.001
Type C	127 (2.9; 2.4–3.4)	62 (2.1; 1.6–2.7)	40 (3.6; 2.6–4.8)	25 (7.4; 4.8–10.7)	< 0.001
Type ABC	2761 (62.7; 61.2–64.1)	1763 (59.9; 58.1–61.7)	744 (66.2; 63.3–69.0)	254 (74.9; 70.0–79.5)	< 0.001

Covariates with *P* ≤ 0.25 were included in the ordered logistic regression

95% CI, 95% Confidence intervals

^a^Chi-square test

^b,c^Statistical analyses did not include missing values and individuals who preferred not to answer

^†^Reference

**Table 2:** Bivariate analysis of characteristics of participants in the 65- to 74-year-old group in relation to their periodontal status

Variable	Overall	Degree of periodontitis			*P* value^a^
		CAL ≤ 3 mm	CAL = 4–5 mm	CAL ≥ 6 mm	
Total, n (%)	4117 (100.0)	830 (20.2)	1428 (34.7)	1859 (45.2)	
Socioeconomic characteristics					
Sex, n (%)					< 0.001
Female^†^	2050 (49.8)	488 (58.8)	772 (54.1)	790 (42.5)	
Male	2067 (50.2)	342 (41.2)	656 (45.9)	1069 (57.5)	
Missing values					
Area, n (%)					0.002
Urban^†^	1996 (48.5)	443 (53.4)	697 (48.8)	856 (46.0)	
Rural	2121 (51.5)	387 (46.6)	731 (51.2)	1003 (54.0)	
Missing values					
Educational level, n (%)					0.198
≤ 9 years^†^	3897 (94.7)	785 (94.6)	1340 (93.9)	1772 (95.3)	
> 9 years	219 (5.3)	45 (5.4)	87 (6.1)	87 (4.7)	
Missing values					
Household income per capita, n (%)					0.012
Less than RMB 5000/person^†^	1186 (28.8)	196 (23.6)	433 (30.3)	557 (30.0)	
RMB 5000 to RMB 15,000/person	1008 (24.5)	221 (26.6)	328 (23.0)	459 (24.7)	
More than RMB 15,000/person	1097 (26.6)	243 (29.3)	385 (27.0)	469 (25.2)	
Prefer not to answer ^c^	826 (20.1)	170 (20.5)	282 (19.7)	374 (20.1)	
Missing values					
Oral health-related behaviours					
Toothbrushing frequency, n (%)					< 0.001
< Once per day^†^	3370 (81.9)	731 (88.1)	1187 (83.1)	1452 (78.1)	
≥ Once per day	747 (18.1)	99 (11.9)	241 (16.9)	407 (21.9)	
Missing values					
Use of dental floss, n (%)					0.073
No^†^	4176 (94.8)	2761 (93.8)	1087 (96.7)	328 (96.8)	
Yes	230 (5.2)	183 (6.2)	36 (3.2)	11 (3.2)	
Missing values					
Use of a toothpick, n (%)					0.006
No^†^	2076 (50.4)	423 (51.0)	673 (47.1)	980 (52.7)	
Yes	2041 (49.6)	407 (49.0)	755 (52.9)	879 (47.3)	
Missing values					
Frequency of dessert consumption, n (%)					0.895
< Twice a day^†^	866 (21.0)	179 (21.6)	296 (20.7)	391 (21.0)	
≥ Twice a day	3251 (79.0)	651 (78.4)	1132 (79.3)	1468 (79.0)	
Missing values					
Smoking status, n (%)					< 0.001
Never^†^	1537 (37.3)	247 (29.8)	461 (32.3)	829 (44.6)	
Current (former)	2580 (62.7)	583 (70.2)	967 (67.7)	1030 (55.4)	
Missing values					
Alcohol consumption, n (%)					0.010
Ceased^†^	390 (9.5)	68 (8.2)	114 (8.0)	208 (11.2)	
Rarely/never	156 (3.8)	28 (3.4)	49 (3.4)	79 (4.2)	
Daily	3217 (78.1)	675 (81.3)	1142 (80.0)	1400 (75.3)	
Weekly	353 (8.6)	59 (7.1)	123 (8.6)	171 (9.2)	
Missing values ^b^	1 (0)	0 (0)	0 (0)	1 (0.1)	
Systemic diseases associated with periodontitis					
Diabetes, n (%)					0.471
No^†^	3617 (87.9)	724 (87.2)	1247 (87.3)	1646 (88.5)	
Yes	500 (12.1)	106 (12.8)	181 (12.7)	213 (11.5)	
Missing values					
Caries type, n (%; 95% CI)					
Type A	2169 (52.7; 51.1–54.2)	443 (53.4; 49.9–56.8)	820 (57.4; 54.8–60.0)	906 (48.7; 46.4–51.0)	< 0.001
Type B	2464 (59.8; 58.3–61.4)	398 (48.0; 44.5–51.4)	846 (59.2; 56.6–61.8)	1220 (65.6; 63.4–67.8)	< 0.001
Type C	708 (17.2; 16.1–18.4)	70 (8.4; 6.6–10.5)	231 (16.2; 14.3–18.2)	407 (21.9; 20.0–23.8)	< 0.001
Type ABC	3326 (80.8; 79.5–82.0)	616 (74.2; 71.1–77.2)	1178 (82.5; 80.4–84.4)	1532 (82.4; 80.6–84.1)	< 0.001

Covariates with *P* ≤ 0.25 were included in the ordered logistic regression

95% CI, 95% Confidence intervals

^a^Chi-square test

^b,c^Statistical analyses did not include missing values and individuals who preferred not to answer

^†^Reference

Covariates with *P* ≤ 0.25 were included in the ordered logistic regression. The primary outcome was the severity of periodontitis, and CAL ≤ 3 mm was used as the reference category. Table 3 shows the association between the number of DFT and periodontal disease severity in the 35- to 44-year-old group. Adults with periodontitis had a significant association with DFT type B (OR: 1.21, 95% CI 1.17–1.25) and type C (OR: 1.40, 95% CI 1.24–1.56). Table 4 shows the association between the number of DFT and periodontal disease severity in the 65- to 74-year-old group. Elderly people with periodontitis had a significant association with DFT type C (OR: 1.28, 95% CI 1.21–1.35).

**Table 3:** Association between the number of teeth with dental caries and periodontal disease severity in the 35- to 44-year-old group

DFT (mean ± SD)	Degree of periodontitis			Model 1 ^a^	Model 2 ^b^	Model 3 ^c^
	CAL ≤ 3 mm N = 2944	CAL = 4–5 mm N = 1124	CAL ≥ 6 mm N = 339	*P* value and OR (95% CI)	*P* value and OR (95% CI)	*P* value and OR (95% CI)
Type ABC	1.93 ± 2.71	2.42 ± 3.09	2.89 ± 3.39	< 0.001 1.07 (1.05,1.09)	< 0.001 1.09 (1.07,1.11)	< 0.001 1.09 (1.07, 1.11)
Type A	1.52 ± 2.22	1.67 ± 2.32	1.65 ± 2.16	0.042 1.03 (1,1.06)	0.001 1.06 (1.03,1.09)	< 0.001 1.06 (1.03,1.09)
Type B	0.38 ± 1.18	0.69 ± 1.55	1.11 ± 2.08	< 0.001 1.23 (1.19,1.27)	< 0.001 1.23 (1.19,1.27)	< 0.001 1.21 (1.17,1.25)
Type C	0.03 ± 0.28	0.06 ± 0.43	0.13 ± 0.61	< 0.001 1.43 (1.27,1.59)	< 0.001 1.44 (1.28,1.6)	< 0.001 1.40 (1.24,1.56)

95% CI, 95% Confidence intervals

^a^Model 1: DFT was included as the only independent variable in the ordered logistic regression analysis

^b^Model 2: Social economic status, sex, area, education level, and household income per capita were added to Model 1

^c^Model 3: Oral health-related behaviours such as smoking status, tooth brushing frequency, use of dental floss, use of a toothpick, alcohol consumption and diabetes were added to Model 2

**Table 4:** Association between the number of teeth with dental caries and periodontal disease severity in the 65- to 74-year-old group

DFT (mean ± SD)	Degree of periodontitis			Model 1 ^a^	Model 2 ^b^	Model 3 ^c^
	CAL ≤ 3 mm N = 830	CAL = 4–5 mm N = 1428	CAL ≥ 6 mm N = 1859	*P* value and OR (95% CI)	*P* value and OR (95% CI)	*P* value and OR (95% CI)
Type ABC	3.34 ± 4.21	4.07 ± 4.24	4.24 ± 4.35	< 0.001 1.03 (1.02,1.04)	< 0.001 1.04 (1.03,1.05)	< 0.001 1.04 (1.03,1.05)
Type A	1.39 ± 2.12	1.50 ± 2.10	1.10 ± 1.70	< 0.001 0.93 (0.9,0.96)	0.001 0.95 (0.92,0.98)	0.002 0.95 (0.92,0.98)
Type B	1.82 ± 3.37	2.27 ± 3.30	2.71 ± 3.66	< 0.001 1.06 (1.04,1.08)	< 0.001 1.06 (1.04,1.08)	< 0.001 1.05 (1.03,1.07)
Type C	0.14 ± 0.57	0.31 ± 1.03	0.43 ± 1.12	< 0.001 1.26 (1.19,1.33)	< 0.001 1.28 (1.21,1.35)	< 0.001 1.28 (1.21,1.35)

95% CI, 95% Confidence intervals

^a^Model 1: DFT was included as the only independent variable in the ordered logistic regression analysis

^b^Model 2: Social economic status, sex, area, education level, and household income per capita were added to Model 1

^c^Model 3: Oral health-related behaviours such as smoking status, tooth brushing frequency, use of dental floss, use of a toothpick and alcohol consumption were added to Model 2

## Discussion

The present study explored the relationship between different types of caries and periodontal disease severity in middle-aged and elderly people in a national sample in China. We discuss the relationship between the two diseases by age group because the risk factors, susceptibility to both diseases, and especially the number and causes of missing teeth in distinct age groups differ. Because bivariate analyses cannot exclude confounders related to periodontitis, ordered logistic regression models were used. According to the statistically significant OR, we found that type B caries (OR 1.21) and type C caries (OR 1.40) in middle-aged people and type C caries (OR 1.28) in elderly people were significantly associated with periodontal disease severity, as the point estimates were very far from the null value. To the best of our knowledge, this study is the first to analyse the relationship between caries and periodontal disease in a national sample of Chinese adults.

There are some potential limitations to this study that should be addressed. First, due to the cross-sectional data used in this study, the causal relationship between caries and periodontitis could not be determined. Second, we were obligated to identify periodontitis using CAL instead of the definitions of periodontitis, such as that described by the Centers for Disease Control and Prevention and the American Academy of Periodontology (CDC–AAP). Third, treatment of periodontal disease could be a confounder. Fourth, the present study considered only decayed and filled teeth as a caries experience, which may not reflect the actual caries experience, as this definition excludes missing teeth due to caries. Fifth, pairing individuals or teeth in this type of research is ideal, which is a significant limitation of this study.

We found that caries types B and C were positively correlated with periodontitis in the middle-aged group, and only caries type C was positively correlated with periodontitis in the elderly group. Carious lesions of both types B and C involve the root surface. The positive correlation between root caries and periodontitis is consistent with that described in previous studies [1, 17]. In a recent systematic review, the incidence or increment of root caries was reported to be associated with patients with periodontal disease [17]. Al Qobaly et al. found that in England, Northern Ireland and Wales, individuals aged 35 years or older with periodontitis had a high risk of coronal and root caries [1]. Periodontal attachment loss could lead to exposure of the root surface. As a result of poor oral hygiene, root caries commonly present as progressive lesions in patients with periodontitis [23].

We found that caries types A and ABC were not correlated with periodontitis in the middle-aged group, and caries types A, B and ABC were not correlated with periodontitis in the elderly group. All carious lesions of types A, B and ABC involve the crown. To date, to the best of our knowledge, only one study has clearly identified the specific types of caries studied to which we can compare our findings [1]. Al Qobaly et al. found that adults with PD/CAL ≥ 4 mm had a 1.03 rate ratio (RR) for coronal caries, in accordance with our results. We found that adults with periodontitis in the middle-aged group had an OR of 1.09 for caries type ABC and an OR of 1.06 for caries type A, and those in the elderly group had an OR of 1.04 for caries type ABC, an OR of 0.95 for caries type A and an OR of 1.05 for caries type B. Since the point estimates were very similar to the null value, we consider these caries types to be irrelevant to periodontitis. The findings of the present study differ from others where a positive or negative correlation between caries and periodontitis was suggested [2–13]. As suggested by some authors, the possible link between the two diseases may be a result of selection [9, 14]. Although these common diseases share putative social-behavioural factors, such as age and poor oral hygiene, there are differences in their microbiological profiles. In addition, the genetic theory of antagonistic pleiotropy suggests that host genetic factors affect susceptibility to caries and periodontitis [1].

Given the complexity of both caries and periodontitis, when analysing the relationship between the two, a method that can measure the two diseases in a multidimensional manner should be adopted. In addition, longitudinal studies in which the reason why each tooth was lost is studied and paired tests are used are needed. Additionally, to address these two common oral diseases, public health policies should be adopted to promote public awareness of oral health care and develop good oral hygiene habits, such as mastering correct toothbrushing methods, regular flossing, oral examinations and timely dental scaling.

## Conclusions

We found that in China, caries types B and C were positively correlated with periodontitis in the middle-aged group, and only caries type C was positively correlated with periodontitis in the elderly group.

## Data Availability

The datasets analysed in this study are available from the corresponding authors upon reasonable request.

## Abbreviations

CAL: Clinical attachment level
CDC–AAP: Center for Disease Control and Prevention and the American Academy of Periodontology
CPI: Community Periodontal Index
DFT: Decayed and filled teeth
PD: Probing depth
PPS: Probability proportional to size
WHO: World Health Organization
